# Tunnel millisecond-delay controlled blasting based on the delay time calculation method and digital electronic detonators to reduce structure vibration effects

**DOI:** 10.1371/journal.pone.0212745

**Published:** 2019-03-22

**Authors:** Xiaoming Guan, Caixia Guo, Ben Mou, Leilei Shi

**Affiliations:** 1 Department of Civil Engineering, Qingdao University of Technology, Qingdao, P.R. China; 2 Department of Civil Engineering, Tsinghua University, Haidian District, Beijing, P.R. China; 3 Beijing, Municipal Construction Engineering Co., Ltd., Beijing, P.R. China; Zapadoceska univerzita, CZECH REPUBLIC

## Abstract

The reasonable delay time of millisecond-delay blasting using digital electronic detonators can significantly reduce the vibration effects induced by tunnel blasting. This study proposes a method for calculating the delay time for cut holes, easer holes and periphery holes, considering the rocks breaking effect as well as wave superposition theory. And then according to the actual layout diagram of the tunnel holes, the delay time calculation formulas of different holes are put forward. Then the delay times were calculated according to the formulas and applied in the field tests. The velocities, rocks breaking and wave superposition cancellation of the vibration using different delay times are analyzed with digital electronic detonators. Then the optimum delay times of different holes were obtained and applied to New Hongyan tunnel project. The velocity and frequency of the vibration with digital electronic detonators are analyzed, compared with non-electronic detonators. The effects of charge and delay time on the velocity and principal frequency of a blasting seismic wave are discussed. The results indicate that the delay time for the holes must be prioritized to achieve breaking effects in the rock with the simultaneous formation of a new free surface, next considering the wave superposition cancellation. When the delay time of cut holes was 5 ms, the rocks breaking effect and wave superposition cancellation effect both worked well. The velocity of the vibration induced by the cut holes blasting was about only 0.46–0.51 cm/s. When the delay time was 6 ms or much longer, the rocks breaking effect would fail. With regard to the easer holes and periphery holes, the optimum delay time of them were all 5ms. The vertical peak particle velocity was reduced from 2.974 cm/s to 0.901 cm/s with digital electronic detonators. Therefore, the velocity had decreased by 69.70% than non-electronic detonators, which was caused by reducing the single simultaneous explosive charge and setting optimum delay time. The proposed delay time calculation method is demonstrated to be sufficiently accurate and can thus be used as a guideline to reduce tunnel blasting vibrations.

## 1. Introduction

Complex environmental tunnels that pass through dense buildings are constructed using the drilling and blasting method are becoming increasingly popular [1,2,3]. Tunnel blasting vibration often causes damage to surrounding buildings and affects the daily work and lives of nearby inhabitants [4,5,6,7]. Therefore, reasonable measures must be taken to control the blasting vibrations. The main concept of the millisecond blasting technique is to control the detonation time and ignition sequence of detonators to achieve the expected vibration suppression effectiveness [8,9,10]. At present, non-electric millisecond detonators are widely used in tunnel blasting vibration control; however, such detonators have many limitations. Because non-electric millisecond detonators are detonated at the same time with several holes, the blasting vibration can be reduced by reducing the cyclical footage, using small charges and dividing the blasting. However, this approach reduces the construction efficiency and extends the construction period. When using the millisecond blasting technique, the delay time accuracy of non-electric detonators is rather low, with a delay error of approximately ± (10–150) milliseconds, because non-electric detonators achieve millisecond delay by controlling the chemical burning rate [11]. Therefore, blasting with non-electric detonators causes uncertainties and instabilities in blasting vibration control that prevent waveform interference from achieving the desired vibration reduction.

With the development of high-precision digital electronic detonators, fine controlled blasting has been successfully applied to the construction of complex environmental tunnels. Digital electronic detonators achieve accurate millisecond delay by using an integrated circuit chip. The scope of the delay time is approximately 1 ms to 16 s, and the delay error is only approximately 0.1 ms. Digital electronic detonators can accurately achieve time delay ignition according to the needs of the conditions on site. A strong rock-crushing effect can be obtained using digital electronic detonators, and damage to structures near the tunnel induced by tunnel blasting under complex environmental conditions can be reduced [12,13]. Moreover, the use of electronic detonators leads to not only a smaller Excavation Damaged Zone (EDZ) but also a lower degree of rock breakage in the EDZ [14]. The use of digital electronic detonators has led to social, environmental and economic benefits.

When tunnel blasting is performed using digital electronic detonators, the blasting vibration can be effectively reduced by setting a reasonable delay time to ensure millisecond delay blasting. The vibration can be further reduced by using a method in which crests and troughs are superposed onto each other or by using a staggered wave crest (or trough) [15] and trough (or crest) superposition method. The key to reducing the vibration using an electronic detonator is to set the delay time properly. In literature [11], the delay time are given according to the experiences of many projects. U. Langefors [16] proposed a millisecond delay interval Δ*t* = *T*/2(T is the vibration wave period) that allows the majority of borehole vibration to cancel each other out under the invariable blasting vibration cycle and the same vibration waveforms. Hinzen [17] found that when using the linear superposition model to control the vibration, the best results are obtained when the error of the delay time of the detonators is less than 1–3 ms. Mogi [18] and Hoshino [19] proposed a combination delay blasting method based on an electronic initiation system. The optimal delay time could be obtained by simulating the superimposed disturbance of the vibrating waveforms of the blasting holes. Zhang [20] used a typical single hole blasting vibration signal from simulations to obtain the optimal delay time. Aldas [21] developed a monitoring and control system of waveform interference for reducing vibrations based on millisecond delay blasting. The optimal delay time could be derived from different delay times by using the wavelet signal and surface wave propagation velocity to simulate multiple hole blasting under different delay time conditions; in this manner, the waveform interference for vibration reduction was achieved. Ling et al. [22] achieved better millisecond delay intervals by performing a wavelet analysis of the time-energy density and time-frequency conversion technology. The distribution of energy density in all frequency bands of a signal with time is called time-energy density function [23]. The time-energy density analysis based on the wavelet transform has the character of making the abrupt change of signal energy prominent and can effectually identify the blasting moment of short-delay detonators. Then the real time of delay can be ascertained by analyzing the energy distribution of the monitoring signals in millisecond blasting, and the problem mentioned above can be solved successfully. Wei et al. [24] proposed the method of precise delay interference for reducing vibrations. Zhang et al. [25] found that when the millisecond delay intervals are *T*/3 <*Δt* <2*T*/3 (T is the vibration wave period), the two seismic waves can reach interference cancellation to different degrees. Wang [26] demonstrated that the real delay time can be identified by analyzing the peak distribution of the instantaneous energy using the Hilbert-Huang Transform (HHT) related to millisecond blasting. Shi [27] considered the control of the maximum charge amount per delay and the selection of the optimum interval time to reduce the vibration intensity by waveform interference in practice. Based on field experiments, the maximum charge amount per delay and 15 ms delay were proposed for use in the site, yielding a vibration reduction of 24.5%.

The optimum delay time *t* in this paper was first proposed by literature [28] in 1962, which was used for the short delay blasts in quarries. The delay time calculation method in quarries was mainly used to achieve the rock optimum breaking effect by providing the new free surface or the collisions between rocks. Besides, the delay time calculation method of millisecond-delay blasting in quarries could also reduce the blasting vibration effect. There are a lot of similarities between quarries blasting and tunnel blasting, such as the rock emulsified explosive, blasting accessories and drilling machine, aiming at rocks optimum breaking effect and reducing the blasting vibration effect. Therefore, the calculation method of delay time in quarries are applied and further developed in tunnel blasting.

In the tunnel blasting, the holes are composed of cut holes, easer holes and periphery holes. The calculation method of delay time is supposed to determine according to the types of holes. Firstly, the importance of the breaking effect is emphasized for the holes. Considering the rocks breaking effect of holes, the method of delay times in quarries blasting are used for tunnel blasting. Next, the wave superposition cancellation effect is considered in the calculation of the delay time. The two seismic waves can achieve good interference cancellation when the delay time is half period of the vibration, according to U. Langefors’ theory. The delay times are determined based on both rocks breaking effect and wave superposition cancellation effect. Finally, the calculated delay times are checked and obtained by field tests. In one word, the delay time is determined by combination of rock optimum breaking effect, wave superposition cancellation effect and field tests adjustment, and the delay time are calculated including the rock’s physical, mechanical and construction parameters according to the actual blasting holes, which are the novelty in this paper.

In order to determine the optimum delay time of different blasting holes, the holes are firstly classified into two types: 1) cut holes and 2) easer holes and periphery holes. The theoretic calculation methods for above two kinds holes are put forward. And then according to the actual holes layout diagram, considering the rocks breaking effect as well as wave superposition theory, the delay time formulas of different holes are studied. Then the delay times were calculated according to the formulas. The different delay times were checked in the field tests in order to find the optimum time and to prove that the calculation method is feasible. The velocity, rocks breaking effect and wave superposition cancellation of the vibration induced by different delay time tunnel blasting are analyzed. The optimum delay time is determined by theoretical analysis and field tests. Finally, the methods of delay time using digital electronic detonators are applied into the actual tunnel, the velocity and frequency are analyzed comparing with that of tunnel blasting using non-electric detonators. The relationships among the vibration velocities, dominant frequency, single initiation charge and electronic detonator delay time are discussed. This work aims to provide the calculation methods of different holes for design of the tunnel blasting construction and reduction of the vibration effect.

The basic information of the tunnel [29] is as follows. The New Hongyan Tunnel is located in Shapingba-Caiyuanba section, which is an interval tunnel of the Chengdu-Chongqing passenger-dedicated line. The geographical coordinates for the tunnel is East Longitude 106.45°, North Latitude29.53°. The tunnel is 6,699 m long, which mileage is from GDK297+295 to GDK303+994. (GDK stands for the distance kilometers of tunnel rerouting.) The train design speed is about 100km/h, which is double line tunnel. The shallow buried depth is between 10 m and 50 m. The stratum mainly consists of mud stone, sandstone and mud stone intercalated with sandstone, with Ⅴ-grade rock accounting for 29% and Ⅳ-grade rock accounting for 71% of the total. The shallow buried tunnel is an underpass and is located near numerous existing buildings. The buildings are mainly 2- or 3-storey masonry structures. These buildings were mainly built in the 1980s and 1990s. The tunnel blasting vibration was a serious threat to the safety of the aging buildings due to their weak and poor shock resistance.

## 2. Methodology

**Fig 1:**
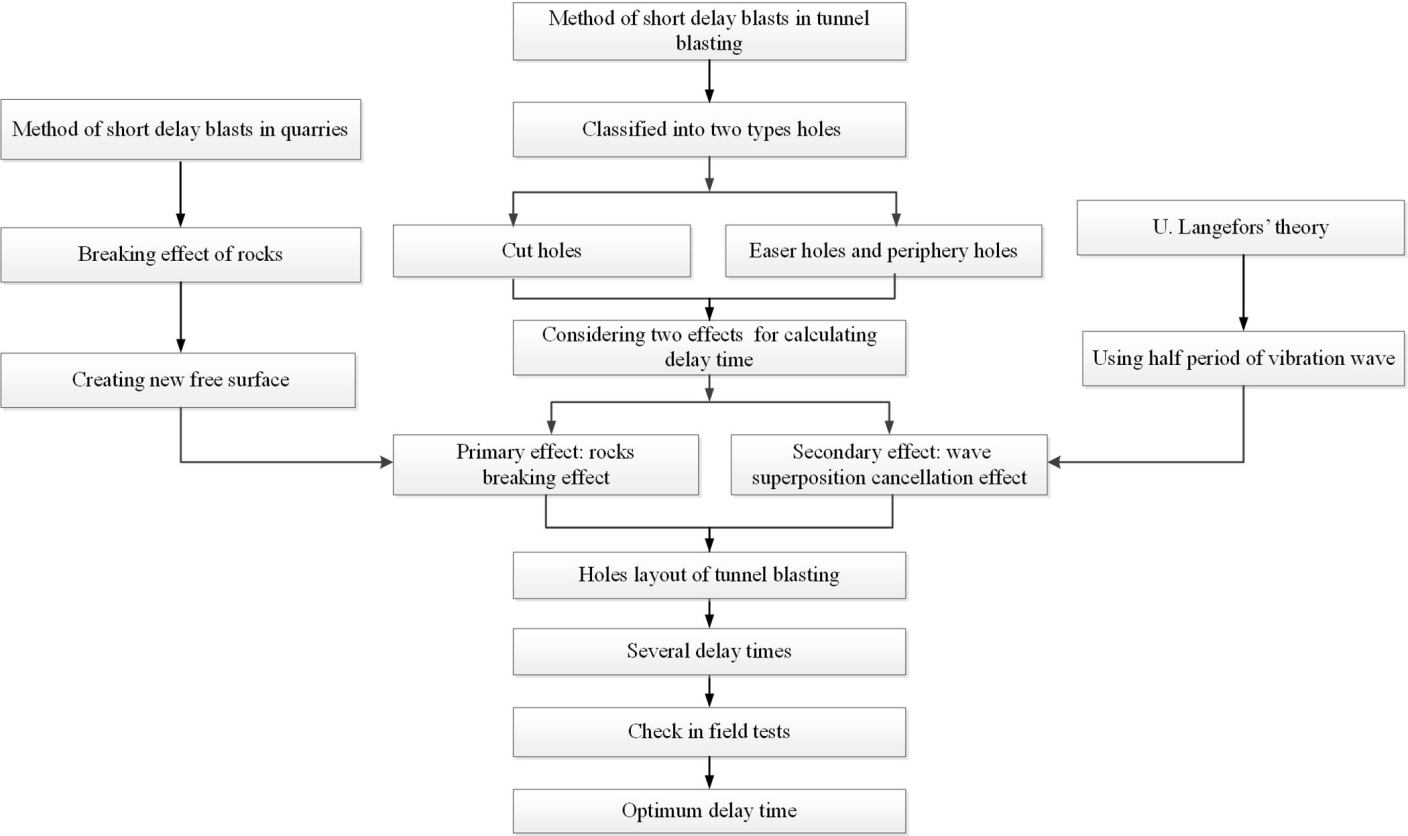
Block diagram of the method of delay time for tunnel blasting.

There are many theories of millisecond delay time calculation for tunnel blasting, such as the method of short delay blasts in quarries considering the breaking effect of rocks [28], the U. Langefors’ theory to reduce the blasting vibration considering the wave superposition cancellation [16], and so on. In the previous researches, the breaking effect of rocks and wave superposition cancellation effect were often considered separately, and above both effects were rarely considered at the same time. Besides the calculation method of delay time of different types holes are not the same. The delay time is supposed to determine according to the types of holes. Therefore, the holes are firstly classified into two types: 1) cut holes, 2) easer holes and periphery holes. Considering the rocks breaking effect as well as wave superposition theory, the theoretic calculation methods for above two kinds holes are put forward. And then according to the actual holes layout diagram and construction parameters, the delay time formulas of different holes are studied. Then the delay times were calculated according to the formulas. The several delay times were checked in the field tests. After analyzing the velocity, rocks breaking effect and wave superposition cancellation of the vibration induced by different delay time, the optimum time could be obtained. The block diagram of the method of delay time for tunnel blasting is given in Fig 1.

### 2.1 Calculation theory of delay time for tunnel blasting

Before cut hole blasting is performed, there is only a free surface in the tunnel. After the cut hole blasting, additional free surfaces for subsequent blasting are successfully created. Thus, the peak particle velocity is mainly caused by the cut hole blasting. The vibration caused by easer holes and periphery holes is often smaller than that resulting from cut hole blasting. Therefore, the key requirement to reducing tunnel blasting vibration is to control the vibration induced by cut hole blasting.

The blasting vibration of cut holes can be reduced through hole-by-hole blasting using digital electronic detonators. The key point is how to set the delay time for the cut holes. If the delay time of hole-by-hole blasting for cut holes is overly long, the breaking effect of rock will fail. Alternatively, if the delay time of hole-by-hole blasting for cut holes is not sufficiently long, several cut holes may initiate simultaneously, leading to a high vibration due to the difficulty of forming new free surfaces and the lack of wave superposition cancellation. Therefore, the delay time of hole-by-hole blasting of cut holes must be calculated considering the breaking effects in the rock simultaneously with the formation of new free surfaces and wave superposition cancellation. Therefore, the delay time for cut holes must be sufficiently long to fully develop the fractures between the first charge and the outline of the rock medium and form a new free surface. Moreover, the delay time for cut holes is based on the fact that the entire rock mass in the vicinity of the first charge is still in a stressed condition to enhance the rock break up when the second charge explodes.

The optimum delay time *t* was first proposed by literature [28] in 1962. According to the findings of his research, the delay time for cut holes is composed of three times and is expressed as follows:

$$t=t_{1}+t_{2}+t_{3}$$ (1)

where, *t*_1_ is the time that the rock medium obtains the stress state induced by the first charge package explosion, that is, the time required for the stress wave to be returned from the charge center to the surface and then from the surface back to the charge center, as shown below.

$$t_{1}=\frac{2w}{c_{p}}$$ (2)

Here, *w* is the resistance line (*m*) and *c*_*p*_ is the longitudinal wave propagation velocity (*m/s*).

**Fig 2:**
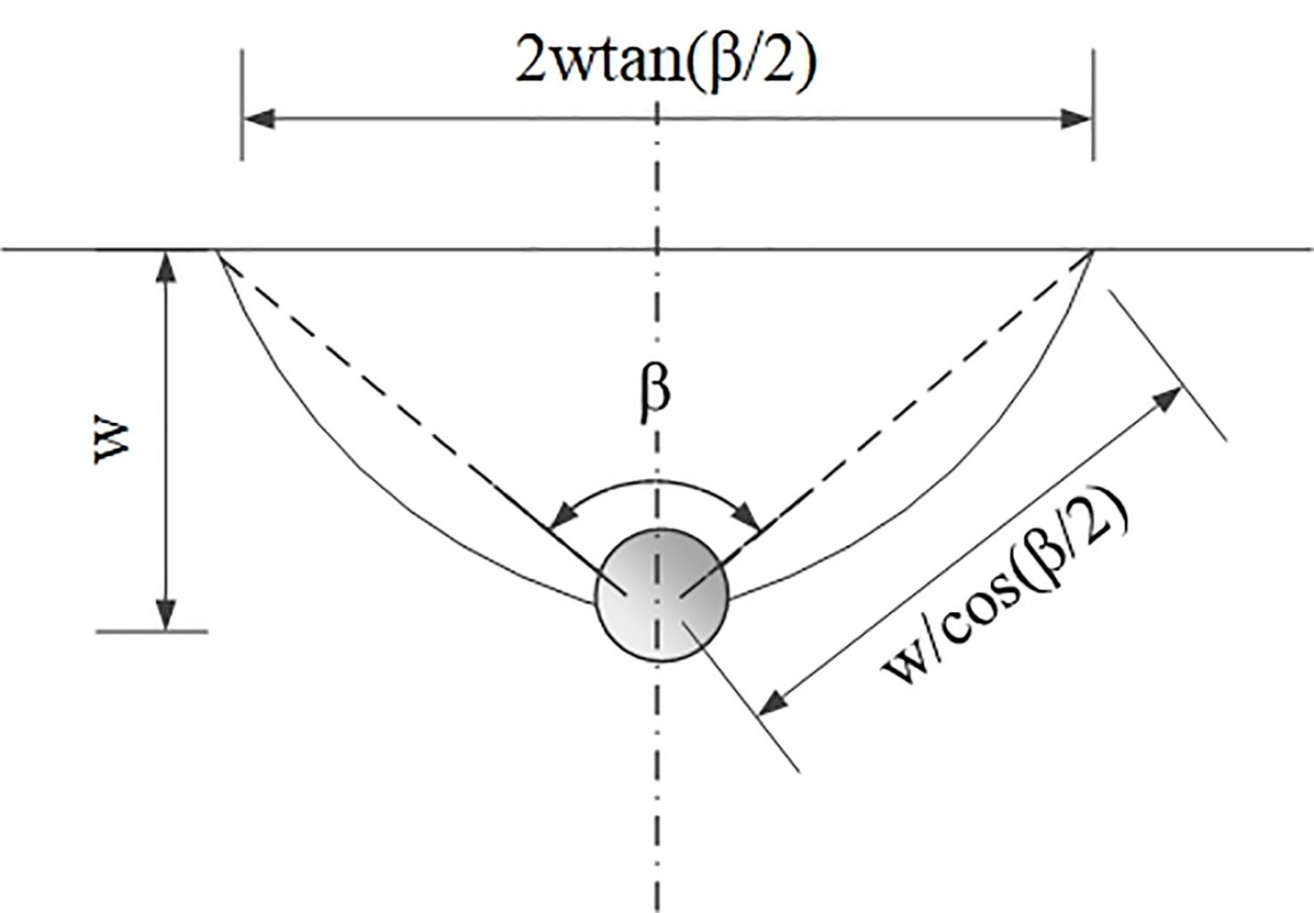
Single-hole blasting casting body.

*t*_2_ is the time from the development of the fracture to the formation of the outline of the thrown rocks on the fracture surface. The shape of the thrown fragments of rocks is different under different conditions. In this paper, we consider a thrown body that is elliptically cut through the hole, as shown in Fig 2; therefore, *t*_2_ can be expressed as

$$t_{2}=\frac{w}{u_{tr}\kappa \cos(\beta /2)}$$ (3)

where *u*_*tr*_ is the rate of propagation of fractures in the homogeneous medium under a unit charge consumption (*m/s*), *κ* is the rupture coefficient of the medium, a constant, and *β* is the angle of the prism of the thrown body (°).

*t*_3_ is the time that the fracture surrounding the prism of the thrown body extends to a sufficient width corresponding to the newly formed free surface. Test results have shown that the width can reach 10 cm while the second cut hole explodes. The pressure in the first cut hole ensures that the rock medium remains in a stressed condition. *t*_3_ can be expressed as

$$t_{3}=\frac{T_{3}w^{2}\rho \tan(\beta /2)}{\phi }=\frac{T_{3}\rho S}{\phi }$$ (4)

In Eq (4), the coefficient *T*_3_ accounts for the effects of certain parameters not present in the equation that appear in the equation of motion; as a first-order approximation, *T*_3_ = 8×10^−5^, a constant. This parameter can be determined more precisely by performing blasting tests. *ϕ* is the hole diameter (*cm*), *ρ* is the density of the rock (*g/cm*^*3*^) and *S* is the area of the thrown body (*m*^*2*^).

Because additional new free surfaces have been formed, the calculation theory of the delay times of blasting of easer holes and periphery holes becomes different from the delay times of the cut holes. The delay time *t*' of easer holes and periphery holes must ensure that the rock is broken up and thrown out, described mathematically as

$$t'>t_{1}+t_{2}+t_{3}$$ (5)

However, considering wave superposition theory, the millisecond delay interval becomes *Δt = T/2*. The two seismic waves can achieve good interference cancellation according to U. Langefors’ theory. Therefore, the delay time for the easer holes and periphery holes must satisfy the following condition:

$$t'\leq \frac{T}{2}$$ (6)

Thus, the delay time for the easer holes and periphery holes is as follows:

$$(t_{1}+t_{2}+t_{3})<t'\leq \frac{T}{2}$$ (7)

where *T* is the period of the blasting seismic wave.

In the near-blasting field of the shallow tunnel, the main carrier of the blasting seismic wave is a longitudinal wave (P wave). The period of the P wave is taken as 4 times *ΔT* (*ΔT* is in Eq no 8). When the P wave is considered separately, the period is independent of the distance and is related to the amount of charge and the properties of the rock. The period *T* of the P wave is proportional to $\sqrt[{6}]{W}$ and can be expressed as follows [30]:

$$T=4\Delta T=4^{P}K_{T}\sqrt[{6}]{W}$$ (8)

In Eq no 8, ^*P*^*K*_*T*_ is the periodic constant of the P wave associated with rock properties, a constant, and *W* is the single initiation charge (*kg*).

### 2.2 Calculating formulas of delay time for tunnel blasting

**Fig 3:**
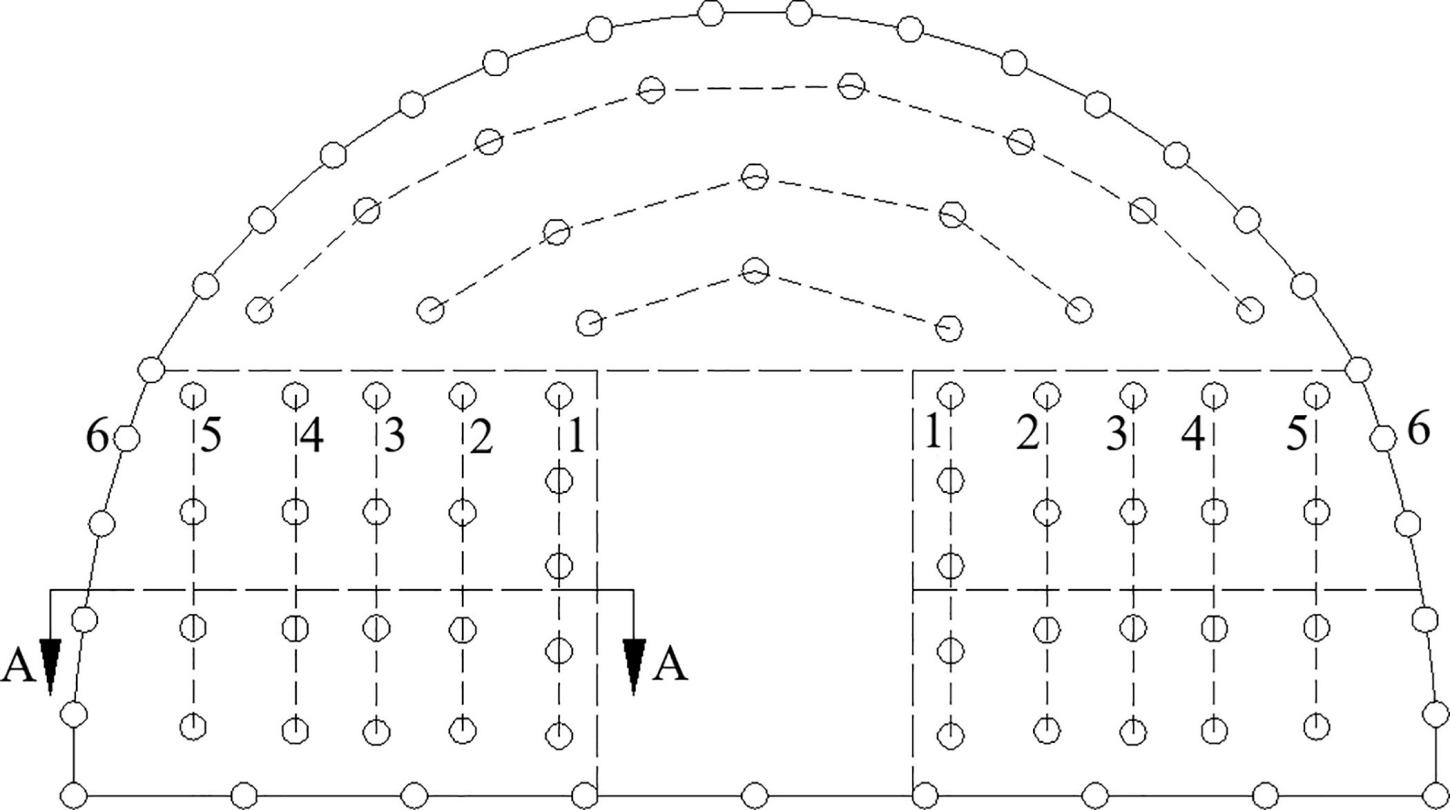
Front view of the wedge-shaped cutting blasting holes.

**Fig 4:**
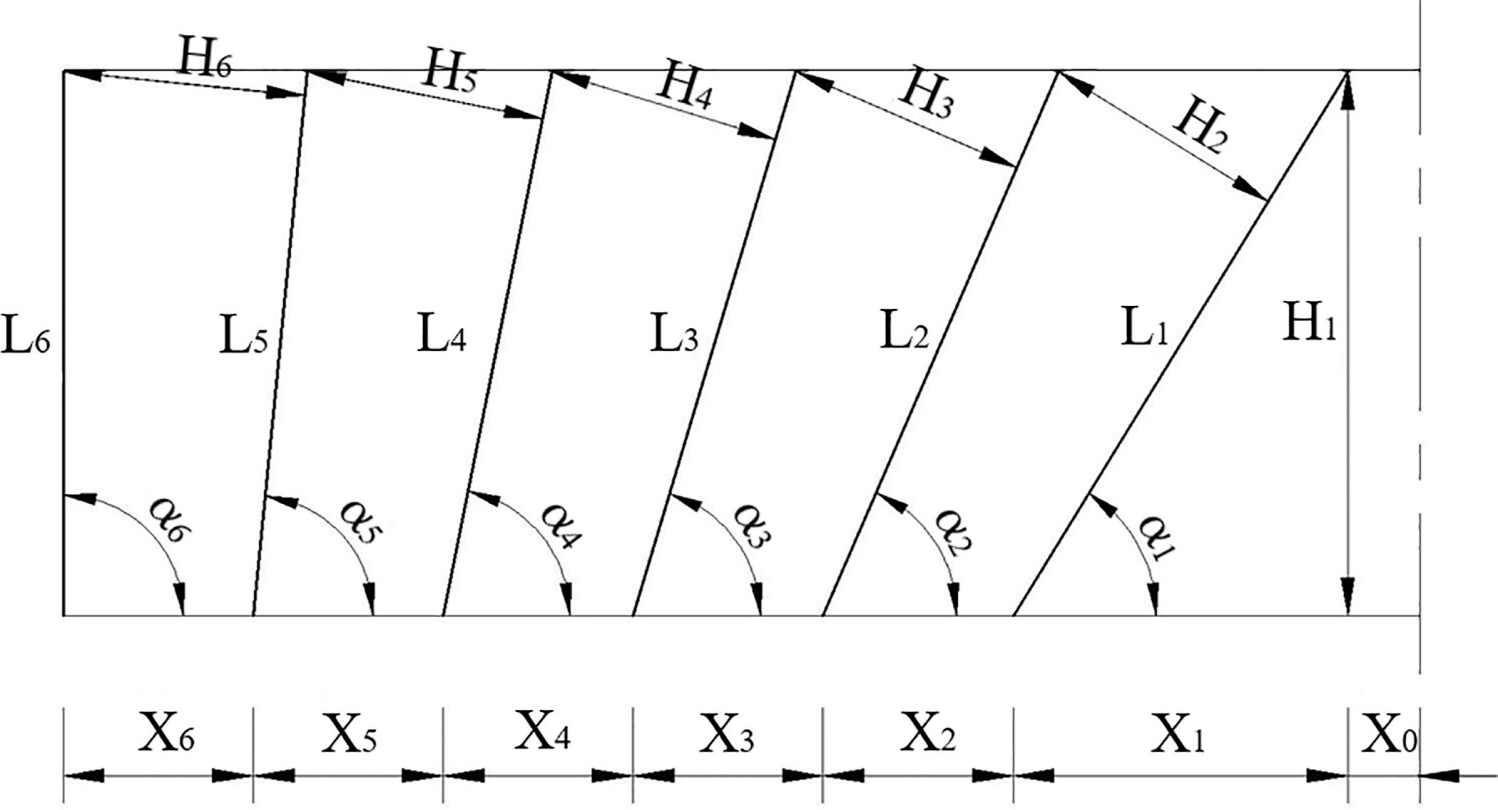
Layout of different blast holes of the A-A tunnel section.

The front view of the wedge-shaped cutting of the tunnel through blasting holes is shown in Fig 3. The layout of different blast holes for section A-A is shown in Fig 4 to illustrate the symmetry of the tunnel. For example, for the blast hole layout shown in Fig 4, a reasonable delay time for blasting the first row of cut holes, the second to fifth row of easer holes and the sixth row of periphery holes was determined, and the delay time for the arch holes referred to the second to fifth row of easer holes and the sixth row of periphery holes.

In Fig 4, *H*_*i*_ refers to the resistance lines of different rows of holes. The maximum resistance line *H*_*i*_ and the depth *L*_*i*_ of the different blast holes was calculated according to the geometrical relationship between different blast holes; based on their relationship, the reasonable delay time was calculated. The relevant equations used for these calculations are as follows:

$$H_{1}=L$$ (9)

$$H_{2}=\left(X_{2}+X_{1}-\frac{L}{\tan\alpha_{2}}\right)\sin\alpha_{1}$$ (10)

$$\overset{5}{\underset{i=3}{H_{i}}}=\left(X_{i}+\frac{L}{\tan\alpha_{i-1}}-\frac{L}{\tan\alpha_{i}}\right)\sin\alpha_{i}$$ (11)

$$H_{6}=\left(X_{6}+\frac{L}{\tan\alpha_{5}}\right)\sin\alpha_{5}$$ (12)

$$\overset{6}{\underset{i=1}{L_{i}}}=\frac{L}{\sin\alpha_{i}}$$ (13)

where *i* is the row number of the blast holes, and *H*_*i*_ is the resistance line of different rows corresponding to the holes used to calculate the time *t*_1*i*_. *L* is the length of tunnel excavation in feet, and *L*_*i*_ represents the depth of different rows of holes used to calculate the time *t*_2*i*_. *X*_0_ is the distance from the bottom of the cut hole to the center of the section, *X*_1_ is the horizontal distance between the bottom and the end of the cut hole and *X*_2_- *X*_6_ are the distances between the blasting holes, as shown in Fig 4. *t*_1*i*_ and *t*_2*i*_ can be expressed as shown below.

$$t_{1i}=2\frac{H_{i}}{c_{p}}$$ (14)

$$t_{2i}=\frac{L_{i}}{u_{tr}\kappa }$$ (15)

By calculating the volume and weight of the discarded rock, the time *t*_3*i*_ was calculated as follows:

$$t_{3i}=8\times 10^{-5}\times \frac{\rho S_{i}}{\phi }$$ (16)

$$S_{1}=(2X_{0}+X_{1})\times \frac{L}{2}$$ (17)

$$\overset{6}{\underset{i=2}{S_{i}}}=(X_{i}+\frac{H_{i}}{\sin\alpha_{i-1}})\frac{L}{2}$$ (18)

Adding Eq (14) to Eq (16) to obtain the total time *t*_*zi*_ yields the following equation:

$$t_{zi}=2\frac{H_{i}}{c_{P}}+\frac{L_{i}}{u_{tr}\kappa }+8\times 10^{-5}\times \frac{\rho S_{i}}{\phi }$$ (19)

where *S*_*i*_ is the area of the discarded rock of each hole (*m*^*2*^), *ρ* is the rock density (*g/cm*^*3*^), *ϕ* is the hole diameter (*cm*), and *t*_*zi*_ is the total millisecond delay time for tunnel blasting (*ms*).

Eqs (9)–(19) enable the reasonable delay time of blasting to be calculated for different holes. In practical tunnel blasting engineering, electronic detonators are used to reduce the tunnel blasting vibration effects. Therefore, first, the engineering rock mass test must be used to determine the rock parameters, such as the velocity of the longitudinal wave in the rock *c*_*p*_, the density of the rock *ρ*, the rock propagation velocity in the fracture *u*_*tr*_ and the rock fracture capacity coefficient *κ*. Second, the relevant blasting construction parameters, such as the maximum resistance line *H*_*i*_, the depth of the blast hole *Li*, the distance *Xi* and the diameter *ϕ* of the blasting holes, must be determined. The accuracy of the millisecond delay time is directly related to the accuracy of the geotechnical and construction parameters.

## 3. Field tests and results

### 3.1 Tunnel blasting vibration with non-electric detonators

**Fig 5:**
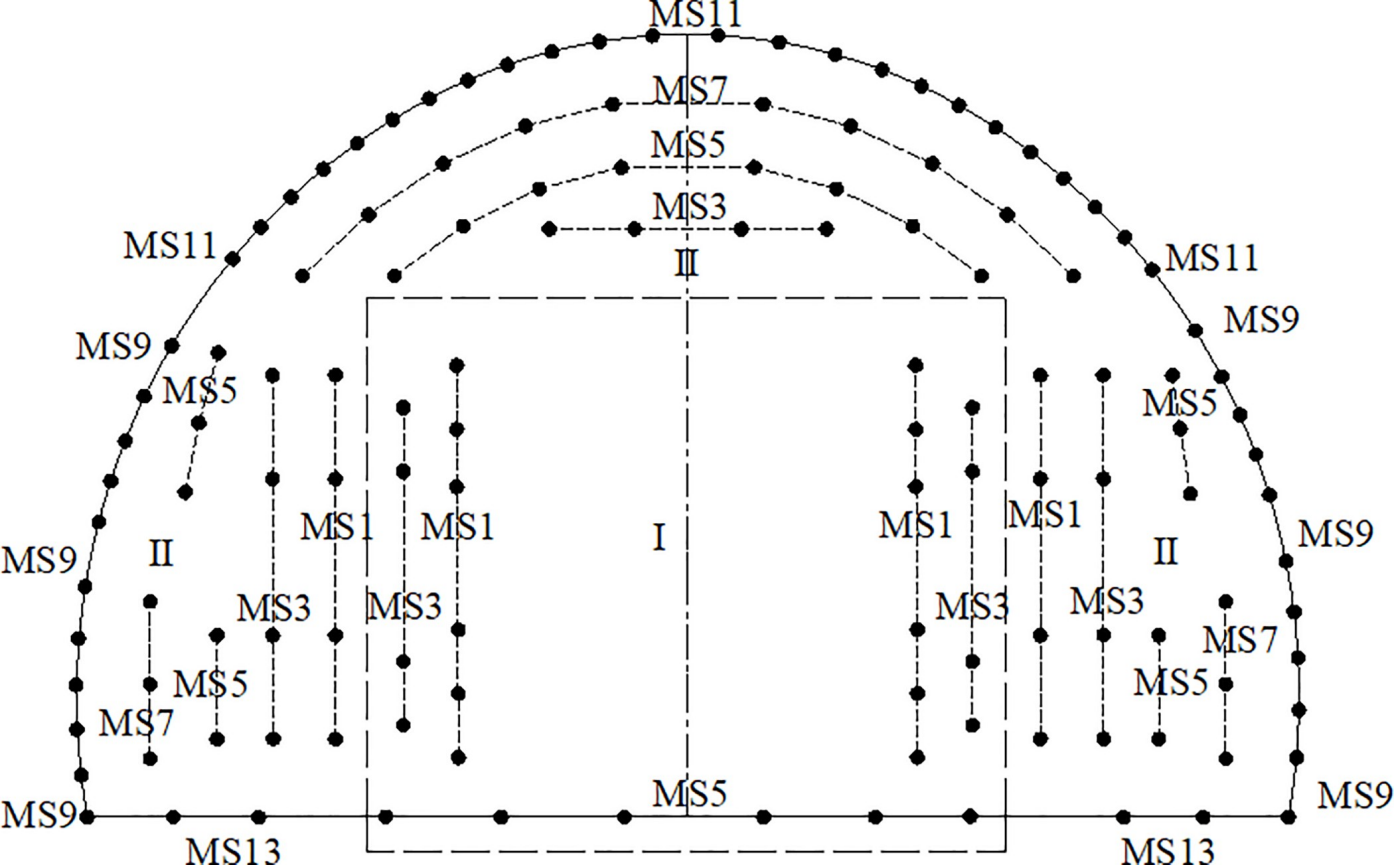
Layout of the blast-holes and the initiation times of non-electric detonators.

**Fig 6:**
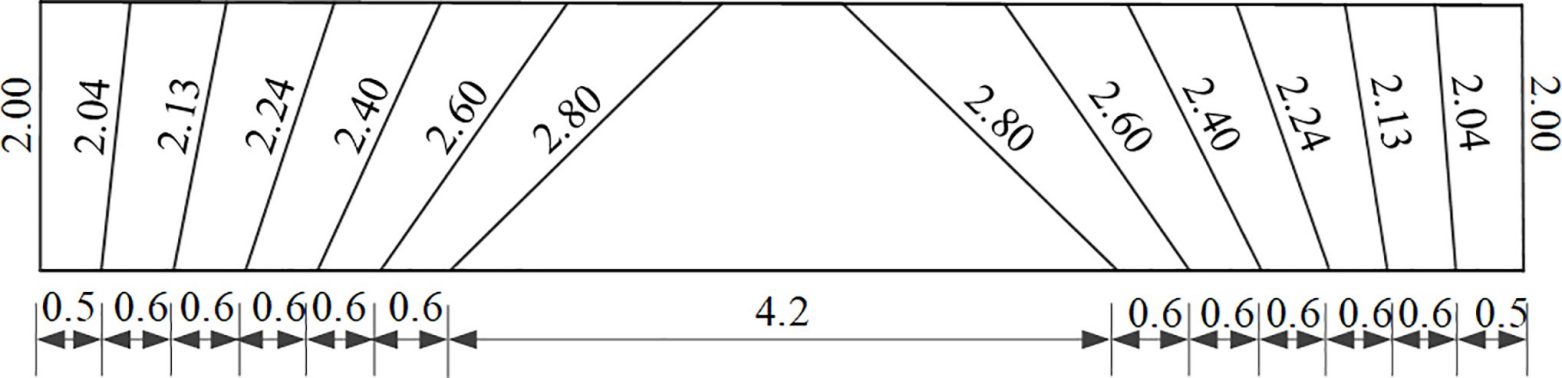
Spacing in drilling and lengths of the holes.

The field tests of tunnel blasting were performed in the New Hongyan Tunnel. The excavation footage of the tunnel was 2 m. The blasting method using non-electric detonators was applied to construct this tunnel, and the total blasting charge was 88 kg. The upper steps were divided into two blasts, I and II, as shown in Fig 5. The layout of the blast-holes and the initiation time are shown in Fig 5. The spacing in the drilling and the lengths of the holes are provided in Fig 6. The blasting parameters using non-electrical detonators is given in Table 1.

**Table 1:** Blasting parameters using non-electrical detonators.

Section	Holes type	Delay time (ms)	Holes length (m)	Number	Single hole charge (kg)	Segment explosive quantity (kg)
Ⅰ	cut holes	MS1	2.80	12	1.2	14.4
	easer holes	MS3	2.60	8	0.8	6.4
	bottom holes	MS5	2.10	6	0.8	4.8
Ⅱ	easer holes	MS1	2.40	8	0.8	6.4
	easer holes	MS3	2.24	12	0.8	9.6
	easer holes	MS5	2.13	18	0.8	14.4
	easer holes	MS7	2.04	16	0.6	9.6
	periphery holes	MS9	2.00	20	0.4	8.0
	periphery holes	MS11	2.00	24	0.4	9.6
	bottom holes	MS13	2.10	6	0.8	4.8

**Fig 7:**
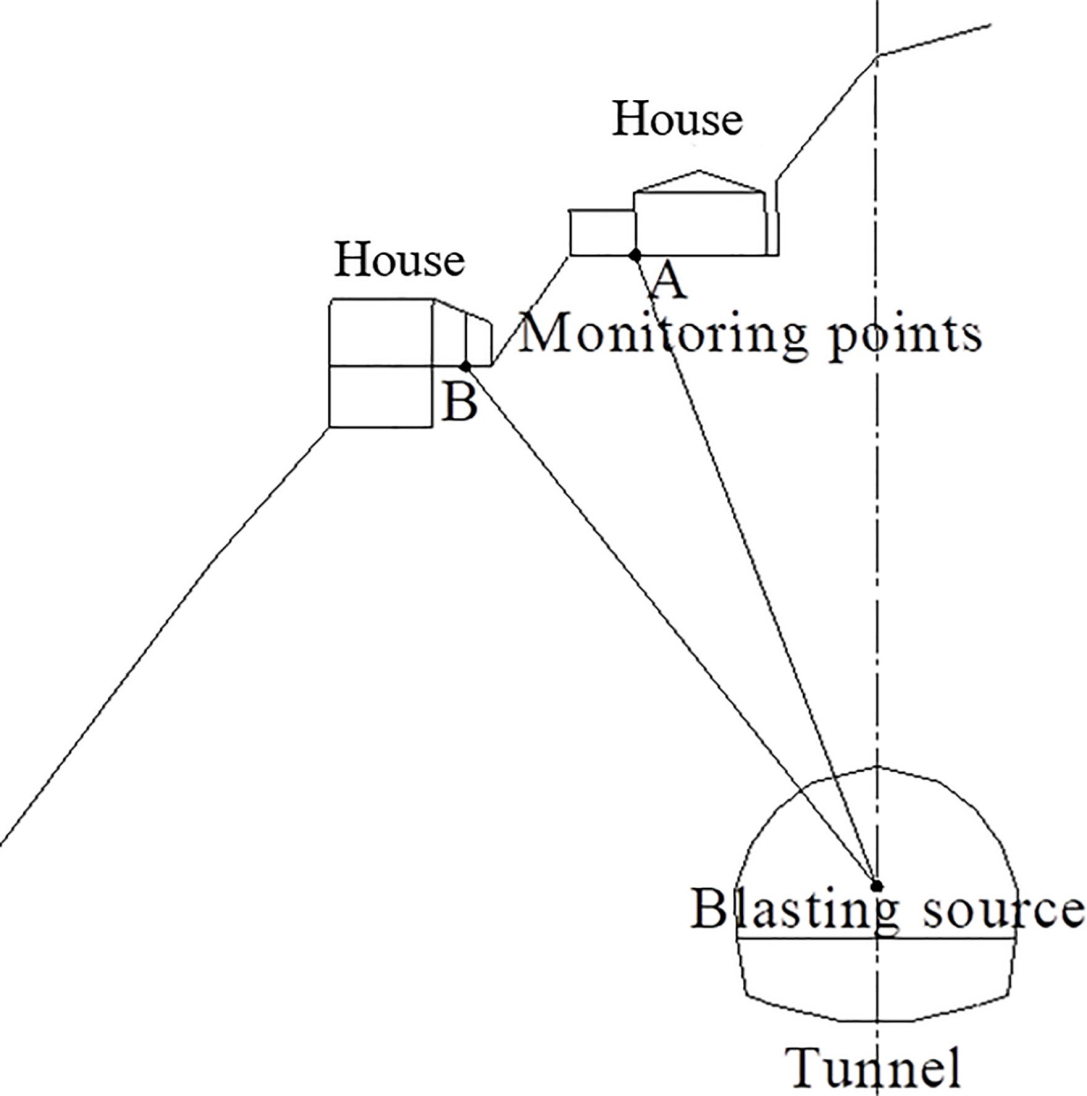
Monitoring point layout of the transverse ground vibration of tunnel.

**Fig 8:**
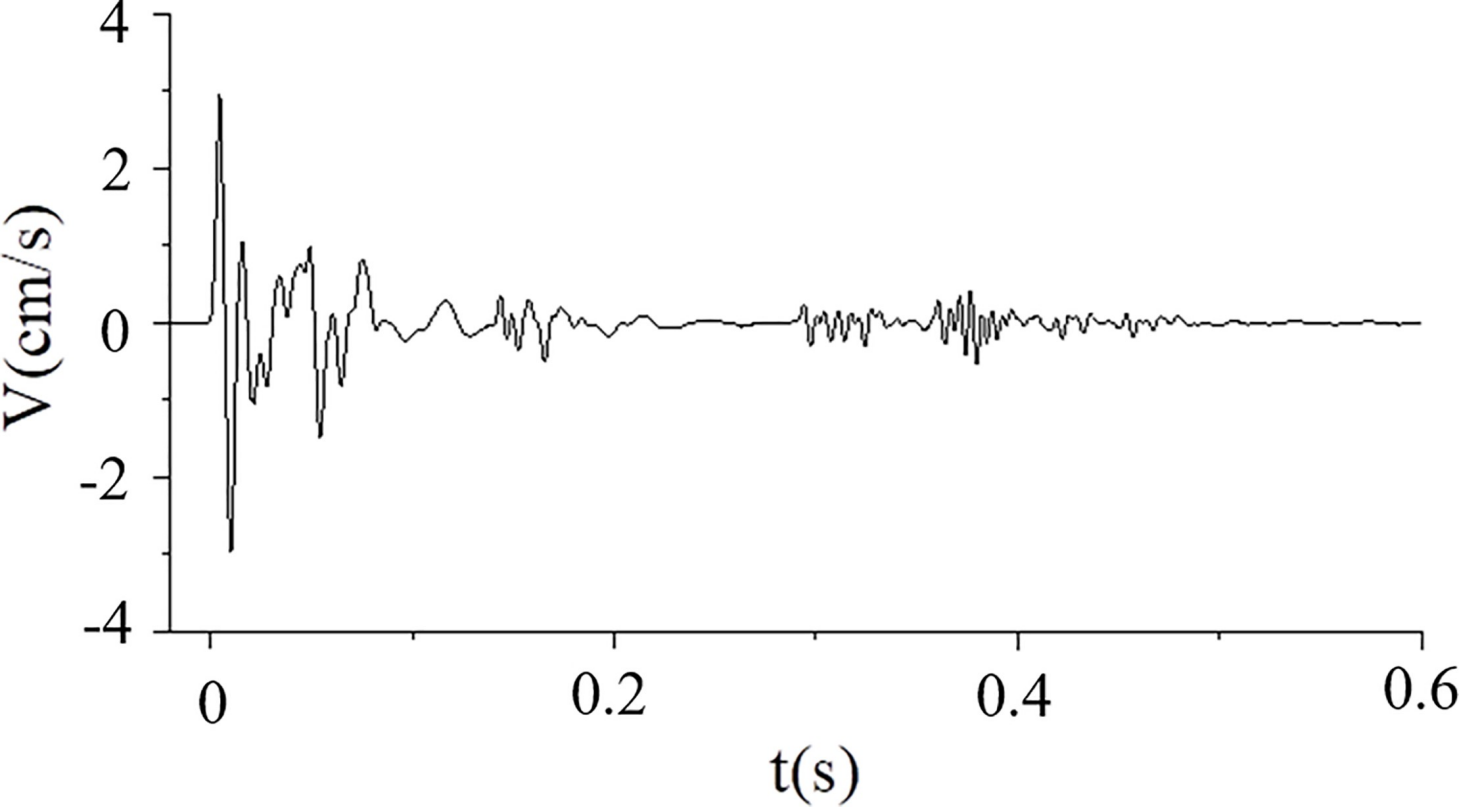
Velocity of tunnel blasting using non-electric detonators (Part I blasting).

**Fig 9:**
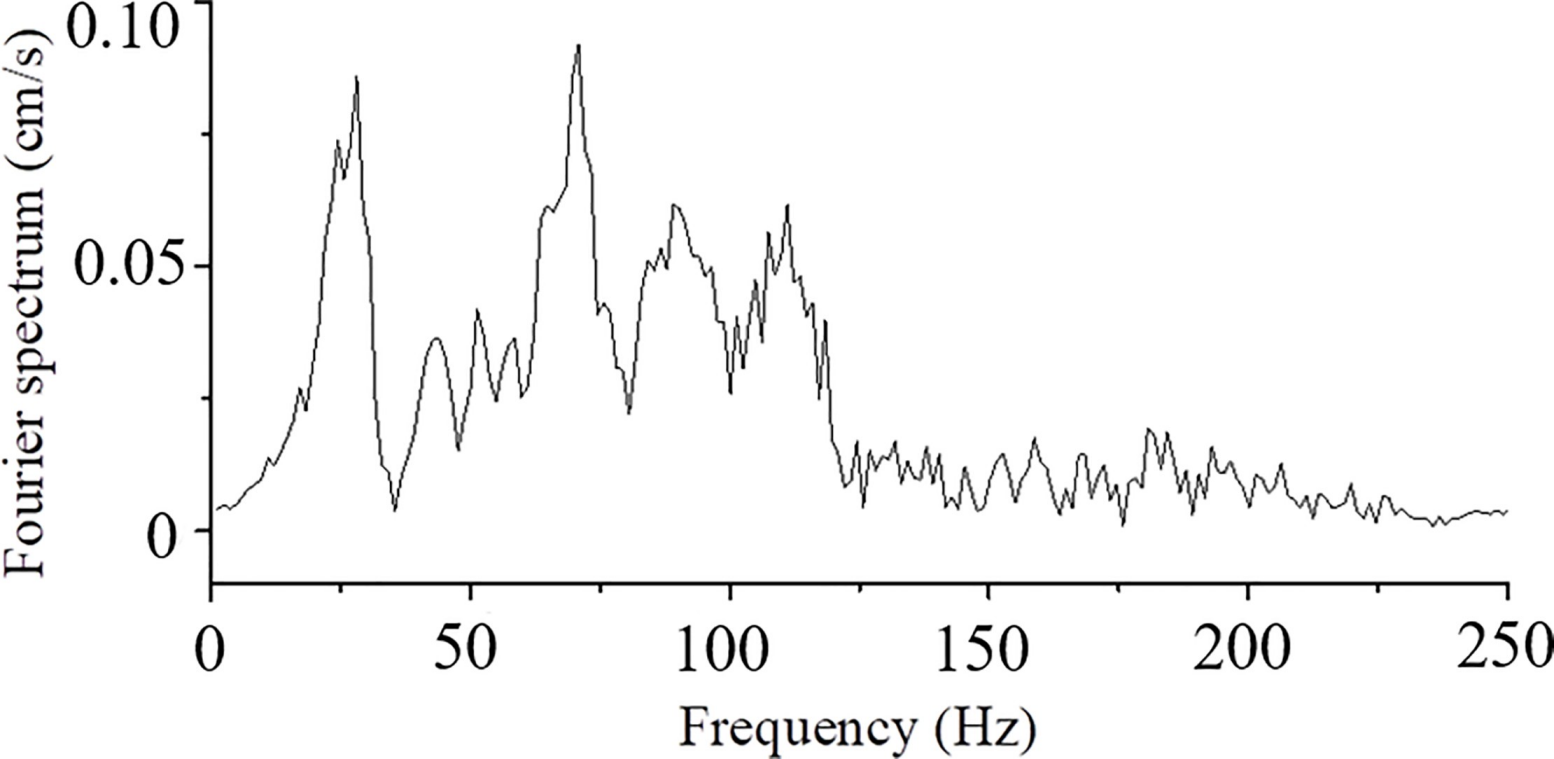
Frequency domain of tunnel blasting using non-electric detonators (Part I blasting).

**Fig 10:**
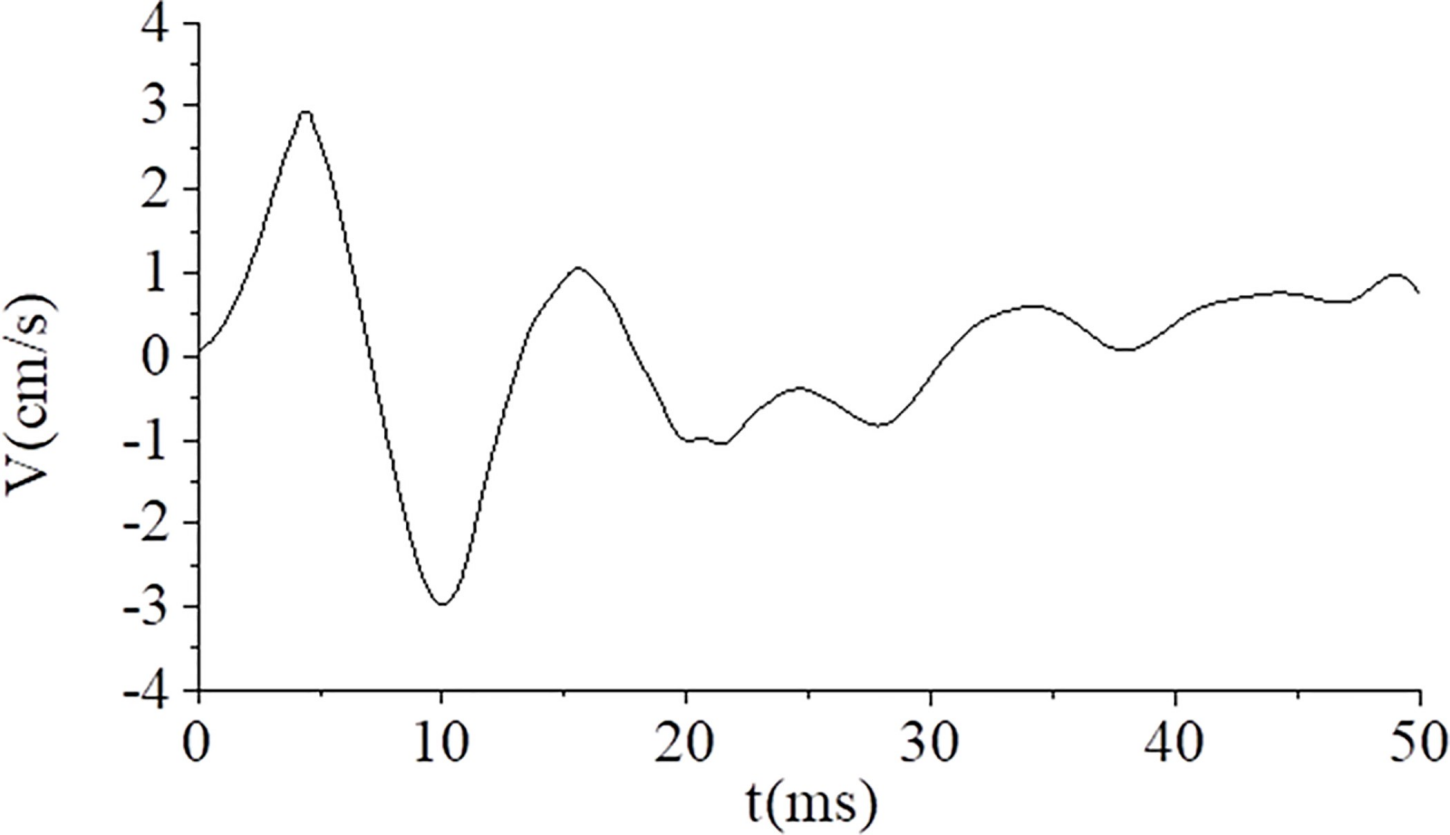
Vibration velocity curve of the blasting signal using non-electric detonators for Part I blasting.

The houses are located above the tunnel. Monitoring points A and B were each monitored using a UBOX-5016 tester made by TDEC Measurement and Control Co. Ltd in Chengdu, China, and the distance from the explosive source was approximately 28.83–29.97 m, as illustrated in Fig 7. The maximum vibration velocities of points A and B measured for Part I blasting using non-electric detonators are shown in Fig 8. The frequency domain of tunnel blasting is illustrated in Fig 9, and the 0–50 ms vibration velocity curve of the blasting signal is shown in Fig 10.

According to Fig 8 and Fig 9, the measured vertical peak particle velocity was 2.974 cm/s, and the principal frequency domain was approximately 25–125 Hz. The vertical peak particle velocity exceeded the safe velocity standard of 2.0–2.5 cm/s for general buildings when the dominant frequency was larger than 50 Hz. In this case, local members may have fractured, in turn seriously threatening the aging buildings around the tunnel. Digital electronic detonators were required to achieve hole-by-hole blasting and reduce blasting vibrations.

Fig 10 shows that the charge of 12 holes blew up simultaneously under only a free surface, and a high peak particle velocity occurred within 14 ms. Furthermore, no wave superposition cancellation effect was observed. After 14 ms, the blasting vibration waveforms began to decay gradually until the blasting of the subsequent holes.

### 3.2 Blasting vibration of cut holes with digital electrical detonators

**Fig 11:**
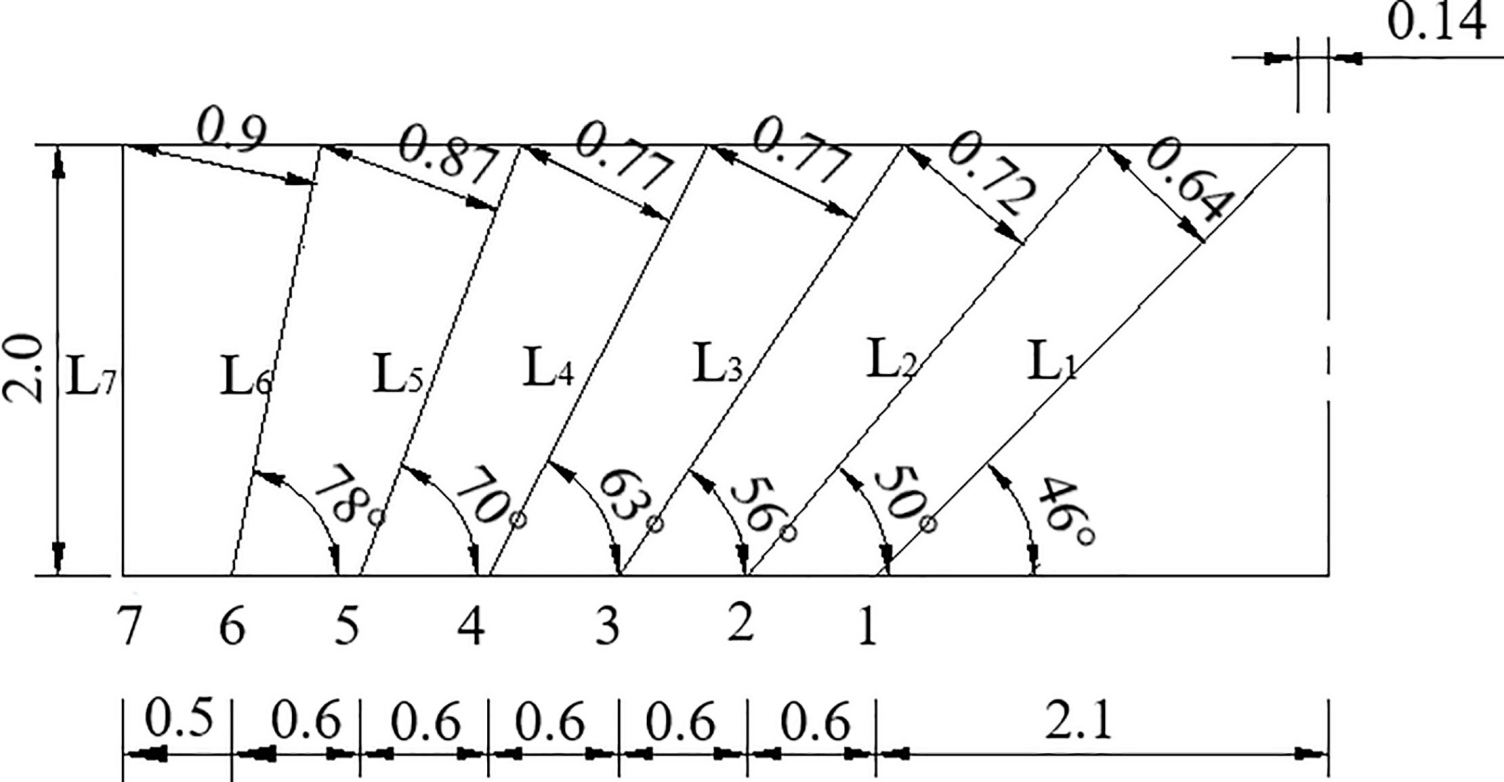
Tunnel holes layout from top view (Unit: m).

The top view of the tunnel hole layout was drawn, as shown in Fig 11, to calculate the relevant construction parameters and a reasonable delay time of milliseconds. Because the tunnel has a symmetrical section, only half of the view was drawn. The position and number of holes are highly similar.

The blasting parameters, such as the blasting hole arrangement and total charge, remained unchanged. The length of the tunnel excavation was 2 m. The considered total number of holes was 130, and the total charge was approximately 88 kg. Among the blast holes, there were 12 cut holes with a single-hole charge of 1.4 kg.

The natural density of rock was measured by means of measuring volume method. The density test results of rock are reported in Table 2, *ρ* = 2.61 *g/cm*^*3*^. The formula is as follows:

$$\rho =\frac{m}{V}$$ (20)

**Table 2:** Density experiment results of sandstone rock.

Number	Diameter (mm)	Height (mm)	Mass (g)	Density (g/cm^-3^)
1	55.23	108.48	672.77	2.59
2	56.87	119.02	776.59	2.57
3	54.59	175.04	1072.84	2.62
4	55.43	116.99	742.10	2.63
5	53.10	188.25	1095.85	2.63
Mean				2.61

In the above equations, *ρ* is the density of the rock, *m* is the mass of the rock and *V* is the volume of the rock.

**Fig 12:**
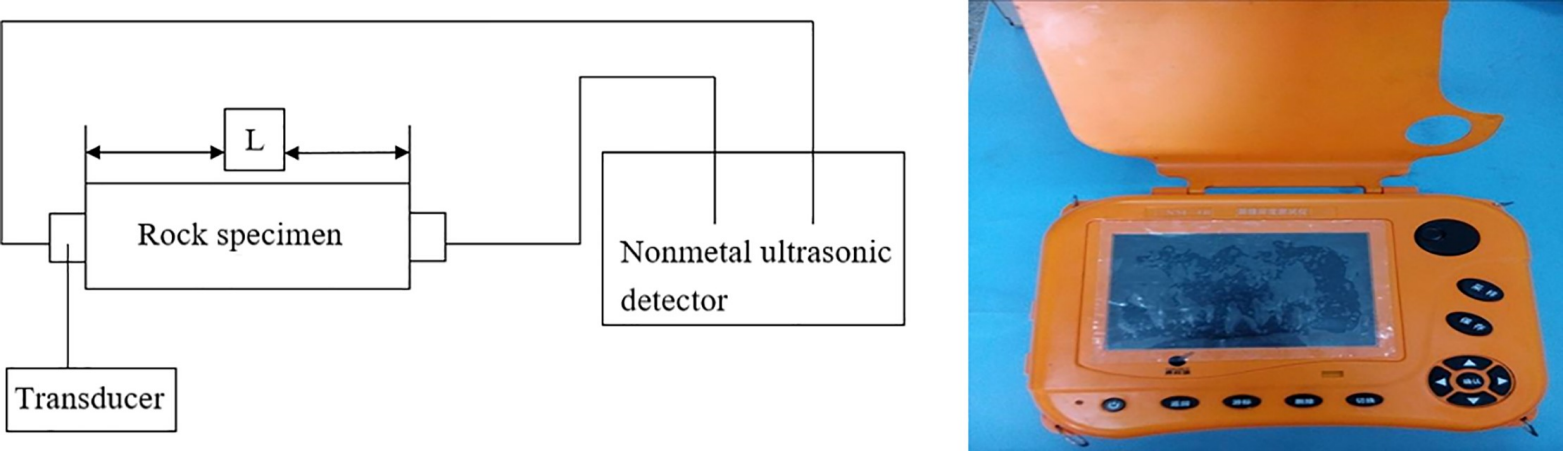
Rock acoustic measurement test and testing instrument.

The velocity of longitudinal wave was obtained by rock acoustic measurement test as *c*_*p*_ = 4500.27 *m/s*. The standard tested specimens were fixed on the test frame. The transmitters set on the ends of tested specimens. In addition, they should cross the center line of tested specimens and measured the distance between the two transmitters. The transmitters should bear 0.05 MPa compression pressure and measure the velocity of longitudinal and transverse wave transmitted in the tested specimens. The rock acoustic measurement test was shown in Fig 12. The test results of rock are reported Table 3. The formula is as follows:

$$V_{p}=\frac{L}{t_{p}-t_{0}}\times 10^{3}$$ (21)

**Table 3:** Velocity of longitudinal wave of the test rock.

Number	L(mm)	T_p_(μs)	T_0_(μs)	V_p_(m/s)
1	108.48	23.5	0	4616.17
2	119.02	26.2	0	4542.75
3	175.04	40.6	0	4311.33
4	116.99	26.0	0	4499.62
5	115.10	25.4	0	4531.50
Mean				4500.27

In the above equations, *V*_*p*_ is the velocity of longitudinal wave of the rock, *L* is the length of the tested rock specimens (*mm*), which is the distance between sending and receiving probes, *t*_*p*_ is the propagation time of the longitudinal wave (μ*s*), *t*_*o*_ is the no time delay of instrument system (μ*s*).

According to the theory of fracture mechanics, the maximum speed of fracture propagation is no more than 0.38 times the compressional velocity [31]. The speed of fracture propagation is approximately *u*_*tr*_ = 1600 *m/s* with *κ* = 0.65. According to the engineering survey data and research reports, *ϕ* = 4.2 *cm*, *S*_1_ is calculated by Eq 17, *S*_1_ = 2.24 m^2^. The millisecond delay times of the cut holes is reported in Table 4.

**Table 4:** Millisecond delay times for cut holes.

Row No.	H_i_(m)	L_i_(m)	S_i_(m^2^)	t_11_(ms)	t_21_(ms)	t_31_(ms)	t_z1_(ms)
1	2.00	2.80	2.24	0.89	2.69	1.11	4.69

**Fig 13:**
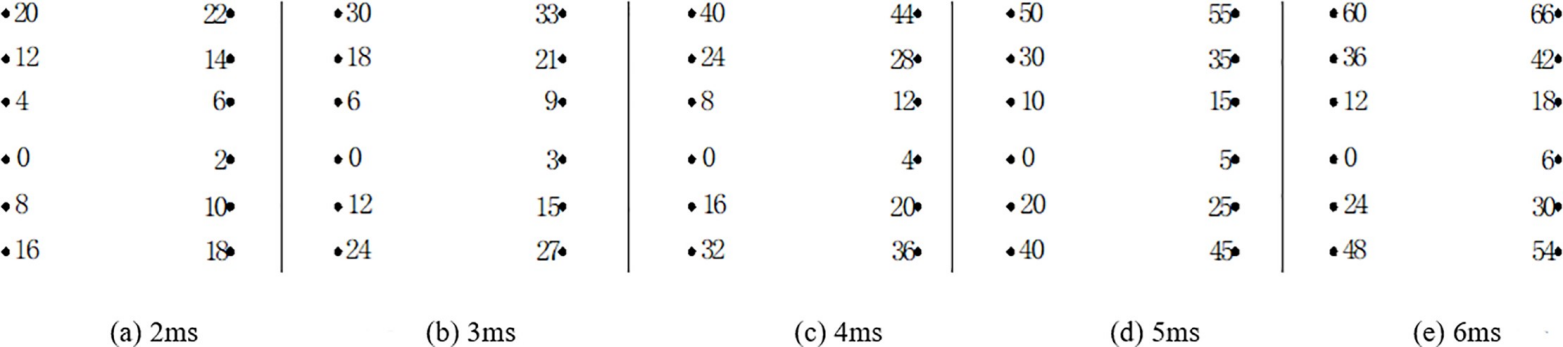
Different delay times for cut holes.

**Fig 14:**
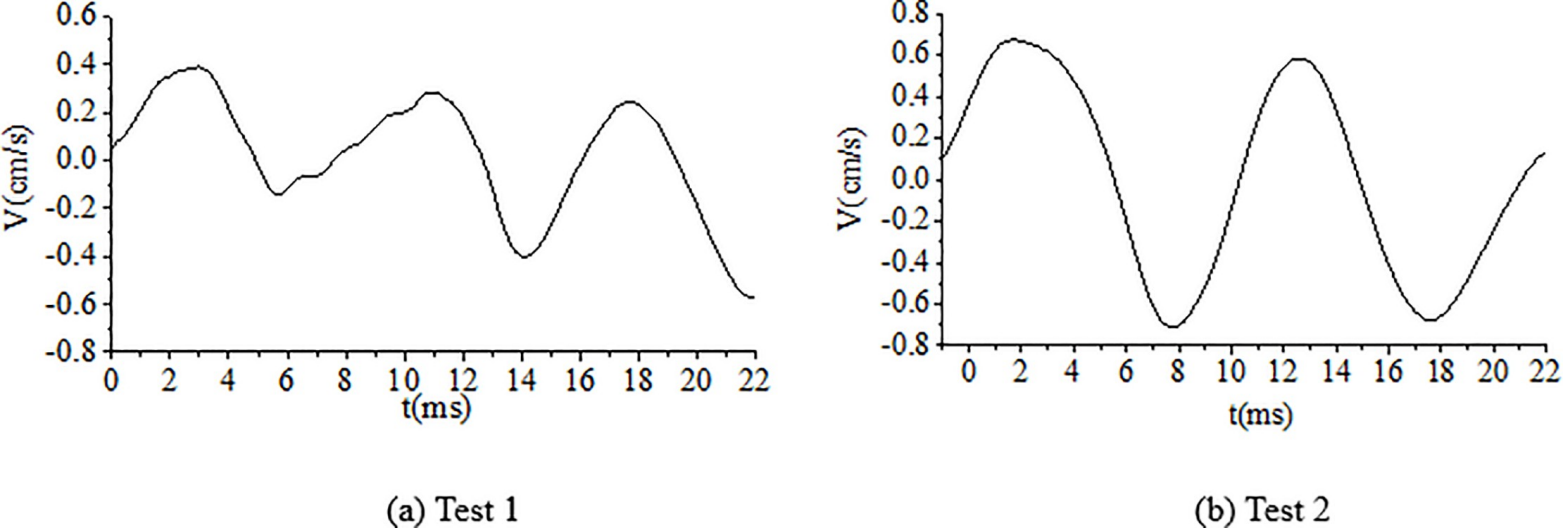
Vibration velocity curves of blasting signals for cut holes for a delay time of 2 ms.

**Fig 15:**
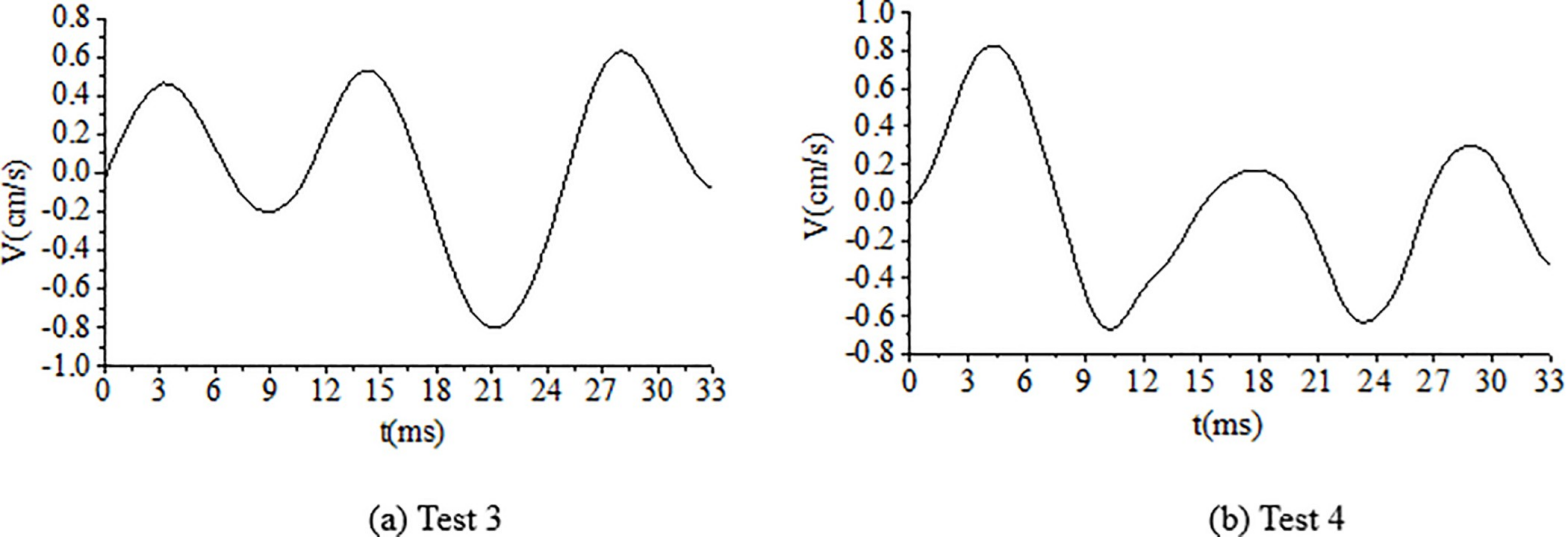
Vibration velocity curves of blasting signals for cut holes for a delay time of 3 ms.

**Fig 16:**
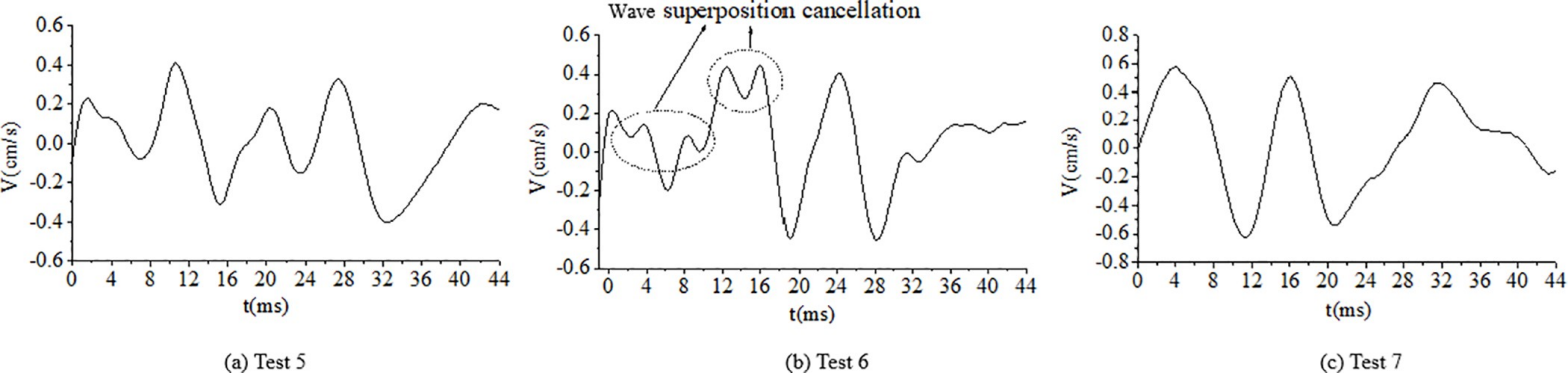
Vibration velocity curves of blasting signals for cut holes for a delay time of 4 ms.

**Fig 17:**
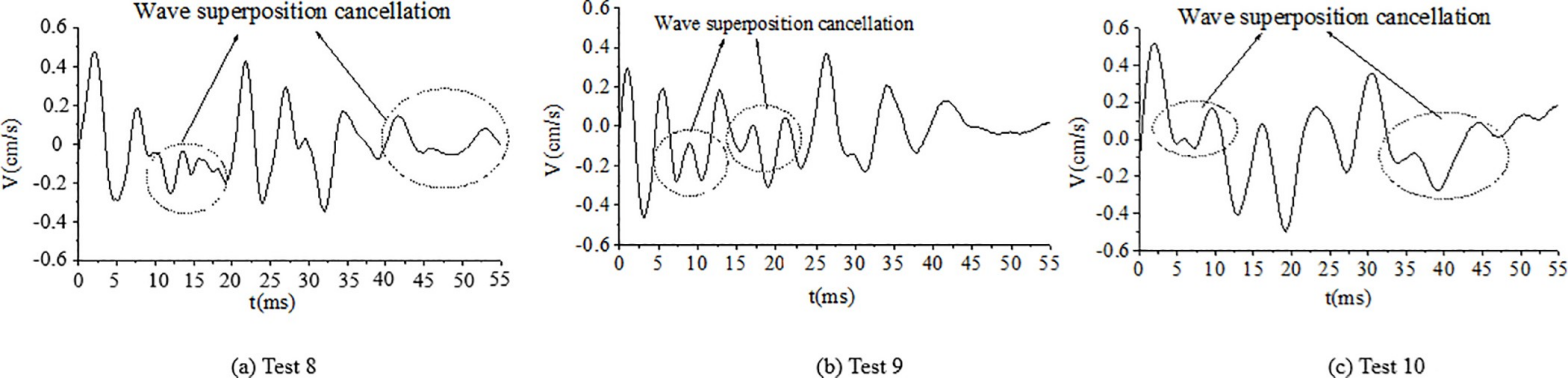
Vibration velocity curves of blasting signals for cut holes for a delay time of 5 ms.

As shown in Table 4, the delay time calculated for the cut holes is 4.69 ms. However, a key point is to estimate the actual time to achieve the breaking effect of the rock simultaneously with the wave superposition cancellation. To determine the optimum delay time, several tunnel blasting tests of different delay times for cut holes, such as 2 ms, 3 ms, 4 ms, 5 ms and 6 ms, were performed in the field using digital electronic detonators. The delay time design of the cut holes is shown in Fig 13. The test results of different delay times for cut holes are reported in Table 5. The result of 6 ms is not provided here because the blasting failed due to an insufficient breaking effect. The typical blasting vibration wave induced by different delay times using the electronic detonators is shown in Fig 14, Fig 15, Fig 16, Fig 17.

**Table 5:** Test results of different delay times for cut holes.

Test number	Delay time (ms)	Total time (ms)	Charge weight per hole (kg)	Distance from explosive source (m)	PPV (cm/s)	Wave superposition cancellation
1	2	22	1.2	32.8	0.58	No
2	2	22	1.2	30.7	0.71	No
3	3	33	1.2	29.7	0.80	No
4	3	33	1.2	28.8	0.82	No
5	4	44	1.2	28.0	0.78	No
6	4	44	1.2	29.7	0.41	Yes
7	4	44	1.2	29.2	0.41	No
8	5	55	1.2	28.5	0.47	Yes
9	5	55	1.2	29.4	0.46	Yes
10	5	55	1.2	28.5	0.51	Yes

As shown in Fig 14, Fig 15, the delay times of 2 ms and 3 ms are considerably less than the calculated delay time of 4.69 ms, the blasting wave induced by digital electric detonator hole-by hole blasting is likely inferior to the non-electric detonator simultaneous blasting wave shown in Fig 10 (0–14 ms). Because of the short delay time, despite several hole-by-hole blasts, the breaking effects in the rock and the formation of new free surface of the adjacent rock did not achieve the desired results at this stage. Therefore, in this case, the simultaneous detonation of several holes eventually yielded undesirable effects in terms of vibration reduction without wave superposition cancellation. This phenomenon was discussed in the first part of section 4.4.

In Fig 16, the delay time of 4 ms is highly similar to 4.69 ms; thus, the breaking effect in the rock simultaneously with the formation of a new free surface in the adjacent blast holes rock occurred at this stage. Furthermore, the influence of wave superposition cancellation occurred in the blasting single, for example, in Fig 16(B). In Fig 17, when the delay time was 5 ms, which is slightly higher than the calculated delay time of 4.69 ms, the fragments of rock existed to ensure the cut holes along with new free surfaces. Moreover, the waves of adjacent blast holes effectively achieved wave superposition cancellation to reduce the vibration. Fig 17 also shows that wave superposition cancellation effect of the blasting wave was remarkable. When the delay time of cut holes was 5 ms, the rocks breaking effect and wave superposition cancellation effect both worked well. The velocity of the vibration induced by the cut holes blasting was about only 0.46–0.51 cm/s. When the delay time was 6 ms or even longer because the adjacent holes were weakened due to the breaking effect. However, the fragments of the broken rocks were not effectively discarded, with less significant free surface of the new rocks. Thus, blasting failure occurred, as has been proved experimentally. In this case, the optimum delay time for the cut holes is 5 ms.

### 3.3 Delay time of easer holes and periphery holes

There are 62 easer holes with a single hole charge of 0.6–0.8 kg; 44 periphery holes with a single hole charge of 0.4 kg and 12 bottom holes with a single hole charge of 0.8 kg.

Regarding the delay time for the easer holes and periphery holes, in addition to calculating the delay time of *t*_1_+*t*_2_+*t*_3_, the period *T* of the P wave also needs to be calculated. According to Eq no 8, for ^*P*^*K*_*T*_ = 0.0032, the period *T*_*a*_ of the easer holes can be calculated as

$$T_{a}=4^{P}K_{T}\sqrt[{6}]{W}=4\times 0.0032\times \sqrt[{6}]{0.6∼0.8}=11.75∼12.3ms$$

$$\frac{T_{a}}{2}=5.88∼6.17ms$$

The period *T*_*p*_ of the periphery holes:

$$T_{P}=4^{P}K_{T}\sqrt[{6}]{W}=4\times 0.0032\times \sqrt[{6}]{0.4}=10.98ms$$

$$\frac{T_{P}}{2}=5.49ms$$

The millisecond delay times of the easer holes and periphery holes are presented in Table 6.

**Table 6:** Millisecond delay times of different blast holes.

Row No.	H_i_ (m)	L_i_ (m)	S_i_ (m^2^)	t_1i_ (ms)	t_2i_ (ms)	t_3i_ (ms)	t_1i_+ t_2i_+ t_3i_ (ms)	0.5T (ms)	t^’^ (ms)	Delay time (ms)
2	0.64	2.6	1.50	0.28	2.50	0.77	3.55	6.17	3.55<t’ ≤6.17	4,5,6
3	0.72	2.4	1.54	0.32	2.31	0.79	3.42	6.17	3.42<t’ ≤6.17	4,5,6
4	0.77	2.24	1.51	0.34	2.15	0.77	3.27	6.17	3.27<t’ ≤6.17	4,5,6
5	0.77	2.13	1.47	0.34	2.05	0.75	3.14	5.88	3.14<t’ ≤6.17	4,5,6
6	0.87	2.04	1.53	0.39	1.96	0.78	3.13	5.88	3.13<t’ ≤5.88	4,5
7	0.9	2	1.42	0.40	1.92	0.72	3.04	5.49	3.04<t’ ≤5.49	4,5

Note: The single-hole charge of rows 2, 3, 4, and 5 is 0.8 kg, and the single-hole charge of rows 6 is 0.6 kg.

The delay time of the easer holes and periphery holes is estimated as 4–6 ms. Combined with the experiment of cut holes in the field, the optimum delay time of the easer holes and periphery holes is 5 ms. The delay time of bottom holes is also 5ms according to the periphery holes.

### 3.4 Reducing tunnel blasting vibration with digital electrical detonators

**Fig 18:**
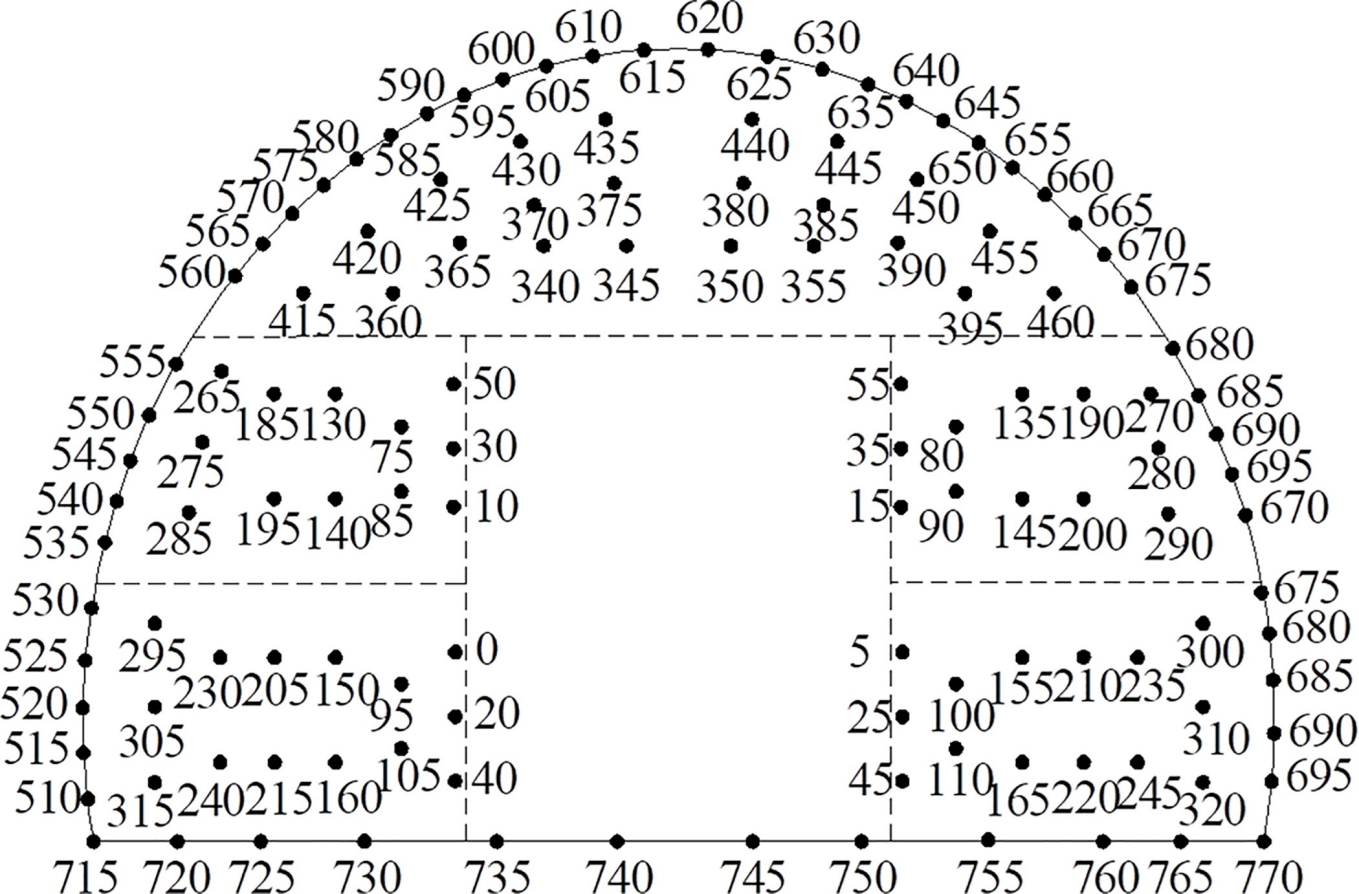
Layout and delay times of the tunnel holes (Unit: ms).

**Fig 19:**
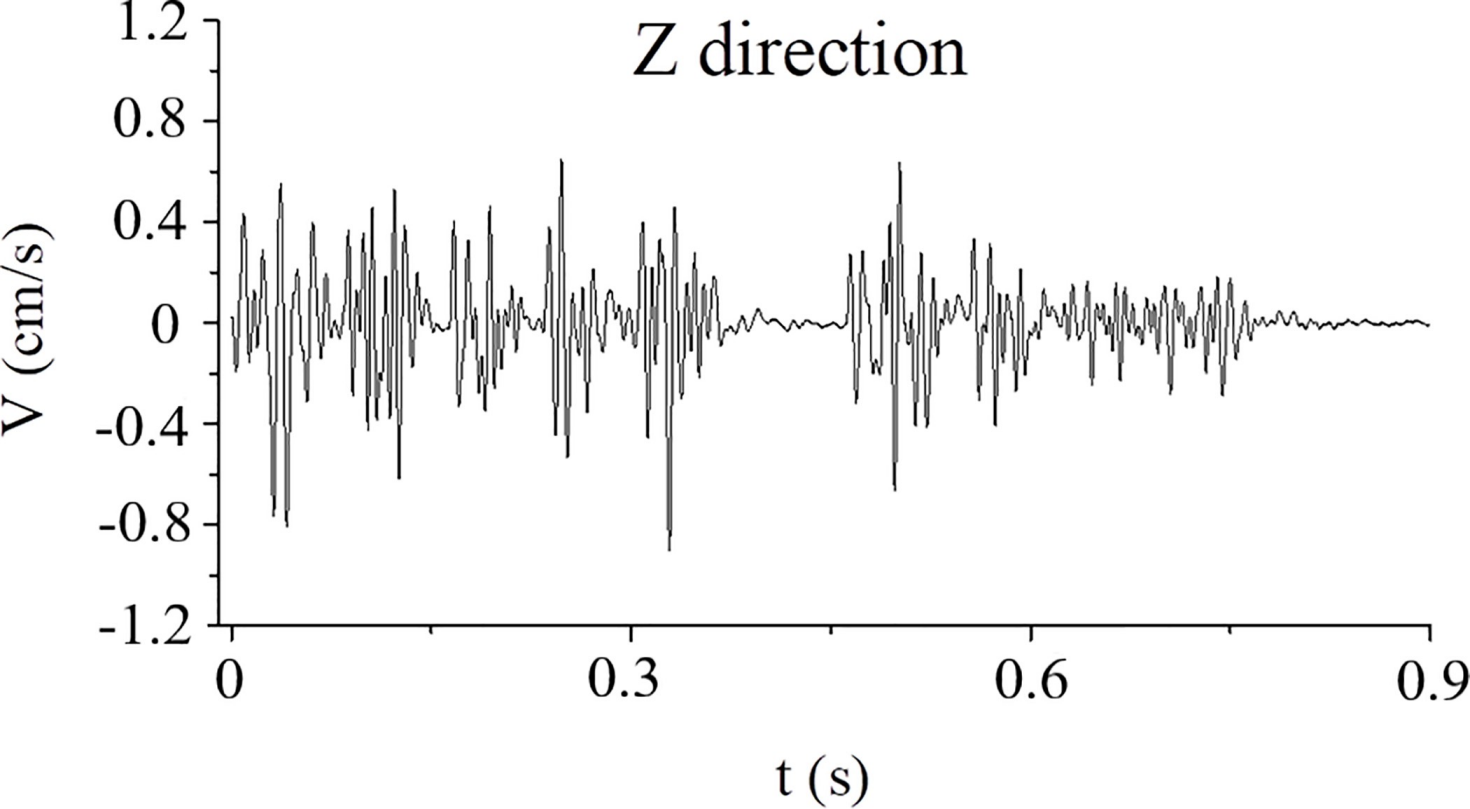
Typical blasting seismic wave using digital electrical detonators.

**Fig 20:**
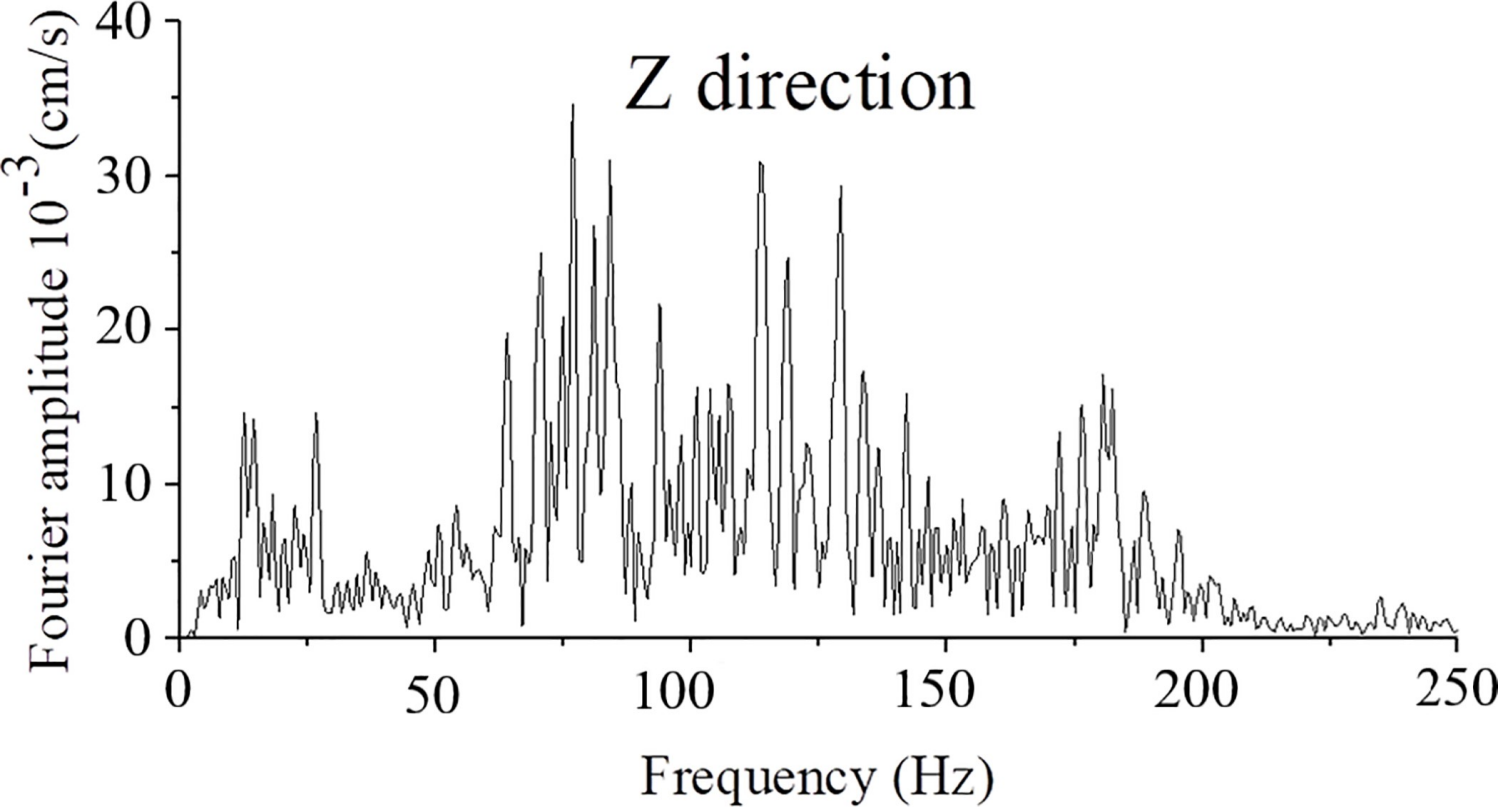
Frequency of tunnel blasting using digital electrical detonators.

The layout of the tunnel blast holes and delay times is shown in Fig 18. The blasting parameters using digital detonators are presented in Table 7. The delay time between rows is approximately 20 ms. The delay time between periphery holes and easer holes is approximately 50 ms (3 to 5 times the vibration periods). The measured vibration velocity is shown in Fig 19, and the frequency domain of tunnel blasting is shown in Fig 20.

**Table 7:** Blasting parameters using digital detonators.

Holes type	Detonation time (ms)	holes length (m)	Number	Single hole charge (kg)
cut holes	0 ~ 55	2.80	12	1.2
easer holes	75 ~ 110	2.60	8	0.8
easer holes	130 ~ 165	2.40	8	0.8
easer holes	185 ~ 220	2.24	8	0.8
easer holes	230 ~ 245	2.13	4	0.8
easer holes	265 ~ 320	2.01	12	0.6–0.8
easer holes	340 ~ 395	2.24	12	0.8
easer holes	415 ~ 460	2.13	10	0.6
periphery holes	510 ~ 695	2.00	44	0.4
bottom holes	715 ~ 770	2.10	12	0.8

From Fig 19, Fig 20, the measured peak particle velocity is 0.901cm/s, and the frequency domain is approximately 75–125 Hz, which not only meets the different criteria of building safety vibration velocity but also ensures that the frequency domain is not similar to the natural frequencies of the buildings. The vertical peak particle velocity was reduced from 2.974 cm/s to 0.901 cm/s with digital electronic detonators. Therefore, the velocity had decreased by 69.70% than non-electronic detonators, which was caused by reducing the single simultaneous explosive charge and setting optimum delay time. The vibration reduction effect using electronic detonators is better than that of ordinary non-electric detonators. The results demonstrate the precision and accuracy of the delay time calculation method.

## 4 Discussion

### 4.1 Influence of the initiating charge on velocity of blasting vibration

In the near-blasting field of shallow tunnels, the P wave is the main carrier of a blasting seismic wave because P wave vibrations have a high frequency and large peak particle velocity. The peak particle velocity of a P wave is proportional to ${\left(\sqrt[{3}]{W}\right)}^{2}$, followed by the S wave, because the propagation velocity of S- and P waves is not significantly different [30]. Furthermore, in the short-range, the S wave charge does not separate from the P wave because the peak velocity of the S wave is proportional to ${\left(\sqrt[{3}]{W}\right)}^{1.7}$ [30]. Therefore, the equations for the peak velocities of the ground surface caused by P and S waves can be expressed as follows [30]:

$$V_{p}{=}^{p}K_{V}{\left(\frac{\sqrt[{3}]{W}}{R}\right)}^{2}(cm/s)$$ (22)

$$V_{S}{=}^{S}K_{V}{\left(\frac{\sqrt[{3}]{W}}{R}\right)}^{1.7}(cm/s)$$ (23)

In the above equations, *V*_*P*_ is the peak velocity of the ground surface caused by P waves (*m/s*), *V*_*S*_ is the peak velocity of the ground surface caused by *S* waves (*m/s*), ^*P*^*K*_*V*_ and ^*S*^*K*_*V*_ are the factors related to the geological blasting method, the geological conditions and other miscellaneous factors, *W* is a charge or detonating charge (*kg*), and *R* is the distance between the measuring point and the center of the blasting source (*m*).

For each particular tunnel, the geological conditions of the rock formation, depth and geological parameters cannot be changed. The blast-holes are initiated using electronic detonators for each hole, with an initiation charge of 1/*n* of the non-electric detonator charge (*n* refers to the number of simultaneous blasting holes). Therefore, the reduction in the peak particle velocity mainly occurs because the digital electronic detonators decrease the charge amount per delay considerably. According to Eqs no 22 and no 23, the millisecond delay blasting method with electronic detonators can effectively reduce the peak velocity of blasting seismic waves.

### 4.2 Influence of the initiating charge on frequency of blasting vibration

As discussed in section 4.1, the main carrier of a blasting seismic wave is the P wave. The period *T* of the P wave is proportional to *W*^1/6^. Furthermore, Meng [32] considered the influence of distance and found that the dominant frequency of a blasting seismic wave *f* changes with the charge and distance as follows:

$$f=\frac{1}{R}{\left(a_{1}\left(\sqrt[{3}]{W}/R\right)+a_{2}R\right)}^{-1}=\frac{1}{a_{1}\sqrt[{3}]{W}+a_{2}R^{2}}$$ (24)

$$T=a_{1}\sqrt[{3}]{W}+a_{2}R^{2}$$ (25)

In the above equations, *α*_1_ and *α*_2_ are constants and *R* is the distance from the center of the blasting.

Without considering the propagation distance of the seismic waves, Eqs no 8 and no 25 provide the same meaning, with a slight difference in the proportional period: in the former, the period is proportional to *W*^1/6^, and in the latter, the period is proportional to *W*^1/3^. The two equations demonstrate that using electronic detonators to achieve hole-by-hole initiation can reduce both the charge of single detonation and vibration wave period and increase the dominant frequency of the blasting vibration wave.

### 4.3 Influence of delay time on velocity and frequency of vibration

The delay time using electronic detonators directly affects the single detonation charge. Therefore, the delay time will affect the peak velocity and vibration frequency of the blasting seismic wave. There are three main situations:

(1) When the delay time of the electronic detonator is set less than the reasonable delay time calculated by Eq no 19, the formation of the split in the rocks and the creation of a new free surface are not complete despite hole-by-hole blasting. This situation is the same as causing several holes to undergo charge detonation simultaneously using non-electric detonators. Because the peak velocity is considerably larger than the single-hole charge detonation and the vibration frequency is less than the single-hole charge blasting, the effects of a reduction in the blasting are not considerable.

(2) When the delay time of the electronic detonator is set longer than 3 to 5 times the vibration period, the tunnel blasting can achieve single-hole charge interval initiation without wave superposition. Each single-hole blasting vibration wave maintains its independence. In this case, the peak velocity and frequency of each hole interval detonation is taken as single-hole charge blasting.

(3) When the delay time of the electronic detonators is longer than the reasonable delay time calculated by Eq no 19 but shorter than 3 to 5 times of vibration periods, hole-by-hole blasting is achieved, and the blasting seismic wave between adjacent holes is superimposed to different degrees. By setting the optimal delay time, the vibration can be reduced by canceling out the crest and trough and avoiding the augmentation of vibration at the crest (or trough).

## 5. Conclusions

This study developed a method for calculating the delay time for tunnel millisecond delay blasting using digital electronic detonators and investigated the influence of charge and delay time on the peak velocity and dominant frequency of the blasting seismic wave through a case study of a tunnel blasting project. The following conclusions are drawn from this research:

(1) The delay time for the holes must be prioritized to achieve breaking effects in the rock with the simultaneous formation of a new free surface, next considering the wave superposition cancellation. The optimum delay time of cut holes was 5 ms, and the rocks breaking effect and wave superposition cancellation effect both worked well. The velocity of the vibration induced by the cut holes blasting was about only 0.46–0.51 cm/s. The optimum delay time of the easer holes and periphery holes were all 5ms.

(2) The method of calculating the delay time was applied in New Hongyan tunnel with digital electronic detonators. The vertical peak particle velocity was reduced from 2.974 cm/s to 0.901 cm/s with digital electronic detonators. Therefore, the velocity had decreased by 69.70% than non-electronic detonators.

(3) Through the incorporation of the results from the field test, the delay time calculation method for tunnel blasting using electronic detonators achieved desirable results in reducing vibrations induced by tunnel blasting. Furthermore, the accuracy and credibility of the electronic detonator delay calculation method for tunnel blasting were confirmed.
